# Two Multiplex PCR Methods for Detecting Several Pathogens Associated with Feline Respiratory and Intestinal Tracts

**DOI:** 10.3390/vetsci10010014

**Published:** 2022-12-26

**Authors:** Xiangyu Xiao, Xiangqi Hao, Bo Chen, Pei Zhou, Shoujun Li

**Affiliations:** 1 Guangdong Provincial Key Laboratory of Comprehensive Prevention and Control for Severe Clinical Animal Diseases, College of Veterinary Medicine, South China Agricultural University, Guangzhou 510642, China; 2 Guangdong Technological Engineering Research Center for Pets, College of Veterinary Medicine, South China Agricultural University, Guangzhou 510642, China

**Keywords:** multiplex PCR, feline pathogens, simultaneous detection

## Abstract

**Simple Summary:**

Coinfection with multiple pathogens is common among feline respiratory tract and intestinal diseases. In clinical cases, cats may exhibit similar symptoms between respiratory and intestinal disease, making it difficult to accurately identify and distinguish pathogens. This study aimed to establish two novel multiplex polymerase chain reactions (mPCRs) for simultaneous detection of pathogens associated with the feline respiratory and intestinal tracts. Regrading sensitivity, the detection limits for FeKoV, FPV, FeAstV, FCoV, IAV, *C. felis*, FeLV, FHV-1 and FCV were 10^3^, 10^4^, 10^3^, 10^3^, 10^3^, 10^4^, 10^4^, 10^5^ and 10^5^ copies/µL, respectively. Moreover, the specificity of the two mPCRs was high. In summary, the two newly mPCR methods provide available, specific, sensitive and inexpensive tools for detecting pathogens.

**Abstract:**

Respiratory tract and intestinal diseases are common threats to feline health. Coinfection with multiple pathogens is not rare among clinical infectious cases. Rapid diagnosis of these coinfections is of great significance for timely and effective clinical treatment. In this study, two novel multiplex polymerase chain reactions (mPCRs) were established for simultaneous detection of four pathogens associated with the feline intestinal tract (feline coronavirus (FCoV), feline astrovirus (FeAstV), feline panleukopenia virus (FPV) and feline kobuvirus (FeKoV)) and five pathogens associated with the respiratory tract (feline calicivirus (FCV), feline herpesvirus 1 (FHV-1), feline leukemia virus (FeLV), *Chlamydia felis* (*C. felis*) and influenza A virus (IAV)). The results of sensitivity analysis revealed that the detection limits for FeKoV, FPV, FeAstV, FCoV, IAV, *C. felis*, FeLV, FHV-1 and FCV were 10^3^, 10^4^, 10^3^, 10^3^, 10^3^, 10^4^, 10^4^, 10^5^ and 10^5^ copies/µL, respectively. Moreover, the specificity of the two mPCRs was high. When the two mPCRs were applied to clinical samples, the assay worked well. In conclusion, we established two mPCR methods that provide an excellent tool for the diagnosis and monitoring of pathogens associated with the feline respiratory and intestinal tracts.

## 1. Introduction

The health problems associated with respiratory and intestinal tract infections are a great concern in cats. In clinical cases, cats are often infected by multiple pathogens and exhibit similar respiratory or intestinal symptoms, making it difficult to accurately identify and distinguish pathogens.

It is a widely held view that feline upper respiratory tract disease (URTD) is an important cause of morbidity and mortality in kittens [1]. URTD is usually caused by one or more pathogen infections, and sick cats often present with respiratory or ocular symptoms. Common infectious pathogens of URTD are feline calicivirus (FCV), feline herpesvirus 1(FHV-1) and *Chlamydia felis* (*C. felis*) [2]. FCV often causes stomatitis, gingivitis and tongue ulceration [3]. FHV-1 causes infectious rhinotracheitis in cats, while *C. felis* mainly causes pneumonia and conjunctivitis [4,5]. In addition, influenza A virus (IAV) can infect cats under natural and laboratory conditions, causing respiratory diseases in cats [6,7,8,9,10,11]. In clinical cases, coinfection of these pathogens is common [12,13]. Differentiating these pathogens is a challenge because sick cats usually show similar symptoms, such as fever, runny nose and cough. Therefore, the identification of these coinfected pathogens via multiple molecular biological methods can provide guidance for clinical treatment and rational prevention. In addition to these common respiratory pathogens, feline leukemia virus (FeLV) is one of the most harmful pathogens in domestic cats worldwide. FeLV-positive cats often suffer from lymphoma, anemia, leukopenia and immunosuppressive diseases [14]. Therefore, timely monitoring of FeLV is necessary.

Feline panleukopenia virus (FPV) is a crucial pathogen that causes feline diarrhea [15]. In addition, enteric-associated pathogens, such as feline coronavirus (FCoV), feline astrovirus (FeAstV) and feline kobuvirus (FeKoV), are prevalent in cats [16,17,18]. FPV is transmitted through the fecal–oral route and mainly infects kittens at the age of 3~6 months, causing severe intestinal and immunosuppressive diseases [15]. There are two types of FCoV, feline infectious peritonitis virus (FIPV) and feline enteric coronavirus (FECV), which are classified according to pathogenicity. FECV is ubiquitous in cats and produces mild intestinal symptoms [19]. During persistent infection, genetic mutations of FECV lead to highly pathogenic FIPV [20]. Moreover, FeAstV may be common in the feces of cats with and without diarrhea [21]. Specific pathogen-free cats experimentally infected with FeAstV have been shown to develop viral excretion and enteritis for a few days [22]. FeKoV, an emerging virus, has been identified in recent years. Epidemiological surveys show that the rate of FeKoV in cats with diarrhea is significantly higher than that in healthy cats [17]. Therefore, it is necessary to monitor FeKoV to further confirm its relationship with cat diarrhea.

Due to the complexity of pathogens associated with feline respiratory and intestinal tracts, rapid diagnosis and detection of these pathogens are important for disease prevention, epidemiological investigation and clinical treatment. Although there are many multiplex polymerase chain reaction (mPCR) methods for feline intestinal and respiratory pathogens, most of these methods can detect only between approximately two and four pathogens, and emerging pathogens are not covered [23,24,25,26]. To provide strong support tools for the rapid diagnosis and epidemiology of sick cats, in this study, the current mPCR method is improved, and two new mPCRs are established to simultaneously detect five types of pathogens associated with feline respiratory (PAFR, namely, FCV, FHV-1, FeLV, *C. felis* and IAV) and four types of pathogens associated with intestinal tracts (PAIT, namely, FCoV, FeAstV, FPV and FeKoV).

## 2. Materials and Methods

### 2.1. Viruses and Bacterial Strains

The genome of rabies virus was extracted from the Nobivac Rabies vaccine. Escherichia coli (ATCC-29522) and Salmonella enterica (ATCC-14028) are kept in our laboratory.

### 2.2. Primer Design

BioEdit 7.0.9.0 software (Ibis Biosciences, Carlsbad, CA, USA) was used to compare conserved sequences of FCV, FHV-1, FeLV, *C. felis*, IAV, FCoV, FeAstV, FPV and FeKoV. Primers were designed based on conserved sequences using Primer 5.0 software (Premier Biosoft International, Palo Alto, CA, USA). All specific primer pairs for mPCRs satisfied the following requirements: similar annealing temperatures; lack of primer dimer formation, hairpin loop formation or self-complementarity; and minimized degenerate bases. The primers (Table 1) were synthesized by the Beijing Genomics Institute.

### 2.3. Nucleic Acid Extraction and Reverse Transcription and Construction of Standard Plasmids

The RaPure viral RNA/DNA Kit (Magen, Guangzhou, China) was used to extract nucleic acids from clinical samples. The extracted total nucleic acids were used for RNA reverse transcription (RT) using HiScriptⅡ1st Strand cDNA Synthesis Kit (Vazyme, Nanjing, China) and amplification. The reaction system volume of RT was 20 µL, consisting of 10 µL of 2 × RT Mix, 1 µL of random hexamers, 7 µL of total RNA and 2 µL of HiScript Ⅱ Enzyme Mix. Finally, the cDNA was stored at −20 °C.

The reaction system volume of mPCR for PAIT and PAFR was 20 µL, including 10 µL of 2 × Taq Master Mix (Vazyme, Nanjing, China), 1 µL of template, 1 µL of the forward primer (10 mM), 1 µL of reverse primer (10 mM) and 7 µL of ddH_2_O. The single PCR program was as follows: 98 °C for 3 min; 36 cycles of 98 °C for 20 s, 57 °C for 1 min, and 72 °C for 1 min 30 s; and 72 °C for 5 min. The products of PCR were detected with 2% agarose gel electrophoresis. The PCR amplification fragments of FCV (1940 bp), FHV-1 (1014 bp), FeLV (608 bp), *C. felis* (500 bp), IAV (155 bp), FCoV (726 bp), FeAstV (418 bp), FPV (337 bp) and FeKoV (198 bp) were ligated into the pMD 18-T vector (TaKaRa, Dalian, China). The ligation products were transformed and cultured, and then the positive plasmids were extracted. Additionally, the positive test plasmids were sent to the Beijing Genomics Institute (Guangzhou, China) for sequencing.

### 2.4. Establishment of the mPCR Methods

To establish the mPCR methods, two mixtures of plasmid standards were used to verify the optimum annealing temperature and the ratio of each primer. The reaction system volume for PAIT was 20 µL, including 10 µL of 2× Taq Master Mix (Vazyme, Nanjing, China), 1 µL of mixed forward primers (FCoV-F(0.26 µL), FeAstV-F(0.16 µL), FPV-F(0.16 µL) and FeKoV-F(0.42 µL)), 1µL of mixed reverse primers (FCoV-R(0.26 µL), FeAstV-R (0.16 µL), FPV-R (0.16 µL) and FeKoV-R (0.42 µL)) and 1 µL of 10^10^ copies/µL of the mixed template (18-T-FCoV, 18-T-FeAstV, 18-T-FPV and 18-T-FeKoV). The reaction system volume for PAFR was 30 µL, consisting of 15 µL of Taq PCR StarMix with Loading Dye (GenStar, Beijing, China), 1 µL of mixed forward primers (FCV-F (0.13 µL), FHV-1-F (0.10 µL), FeLV-F (0.24 µL), *C.felis*-F (0.19 µL) and IAV-F (0.34 µL)), 1 µL of mixed reverse primers (FCV-R (0.13 µL), FHV-1-R (0.10 µL), FeLV-R (0.24 µL), *C.felis*-R (0.19 µL) and IAV-R (0.34 µL)), 1 µL of 10^10^ copies of the mixed template (18-T-FCV, 18-T-FHV-1, 18-T-FeLV, 18-T-*C.felis* and 18-T-IAV) and 12 µL ddH2O. Both mPCR procedures were as follows: 98 °C for 3 min; 36 cycles of 98 °C for 20 s, gradient annealing temperature of 50~60 °C for 1 min and 72 °C for 1 min; and 72 °C for 5 min.

The products of mPCR (15 μL) were detected with 2% agarose gel electrophoresis.

### 2.5. Specificity of the mPCR Methods

We used a feline kidney cell line (F81, ATCC-CL-0081) and other pathogens (Salmonella enterica, rabies virus and Escherichia coli) as templates to verify the specificity of the mPCR methods. Single plasmids and mixed plasmids were also detected. Furthermore, the negative control for mPCR was empty pMD 18-T vector.

### 2.6. Sensitivity of mPCR Methods

The plasmid mixtures and individual plasmids were diluted in a gradient from 1 × 10^10^ copies/μL to 1 × 10^1^ copies/μL. The diluted plasmids were subsequently used to verify the sensitivity of the mPCR methods.

### 2.7. Examination of Clinical Samples

Twenty-three anal swabs and twenty nasal swabs from cats with respiratory or intestinal disease were collected with the consent of the cat owners. Clinical samples were kept at −80 °C prior to nucleic acids extraction. The template was 1 μL of cDNA obtained by RT. Eventually, each cDNA was used for clinical testing via the mPCR methods.

## 3. Results

### 3.1. Optimum Annealing Temperature for the mPCR Methods

**Figure 1:**
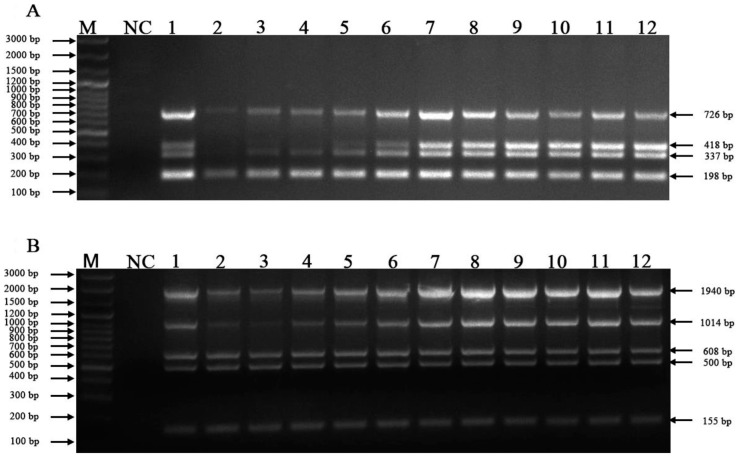
Optimal annealing temperatures for the mPCR methods. (**A**) Optimal annealing temperatures for FeKoV, FPV, FeAstV, and FCoV; (**B**) Optimal annealing temperatures for FCV, FHV-1, FeLV, *C. felis* and IAV. Lane M, DNA marker (100 bp ladder plus); lane NC, negative control; lanes 1~12, 50.0 °C, 50.5 °C, 51 °C, 52 °C, 53.2 °C, 54.4 °C, 55.6 °C, 56.8 °C, 58.0 °C, 59.0 °C, 59.5 °C and 60.0 °C, respectively.

By adjusting the concentration of each primer, annealing temperature, extension time and cycles, the optimal reaction conditions were explored. The mixture of plasmids was applied to verify the mPCR results, and the products of the mPCR were detected with 2% agarose gel electrophoresis. The optimal annealing temperature was 55.6 °C for mPCR of FCoV, FeAstV, FPV and FeKoV (Figure 1A) and 56.8 °C for mPCR of FCV, FHV-1, FeLV, *C. felis* and IAV (Figure 1B), in which no nonspecific amplicons or primer dimers were generated.

### 3.2. Specificity of mPCR Methods

**Figure 2:**
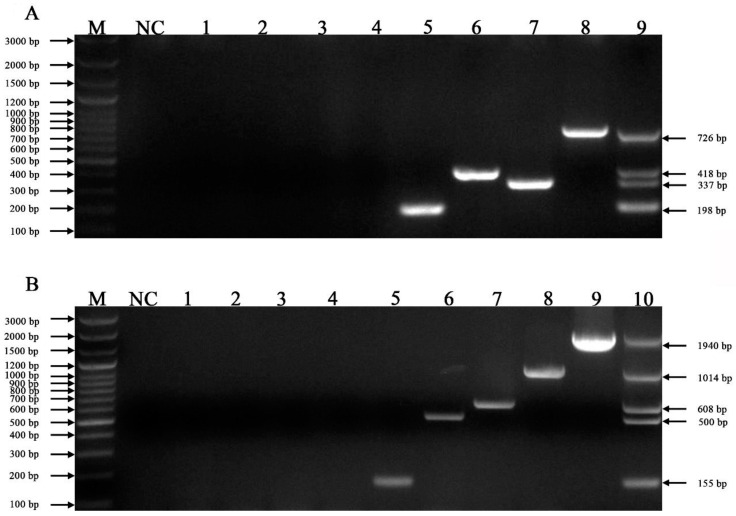
Specificity of the mPCR methods. (**A**) Lane M, DNA marker (100 bp ladder plus); lane NC, negative control; lane 1, F81; lane 2, Escherichia coli; lane 3, Salmonella enterica; lane 4, R rabies virus; lanes 5~8, 18-T-FeKoV, 18-T-FeAstV, 18-T-FPV and 18-T-FCoV, respectively; lane 9, mixed standard of 18T-FeKoV/ 18-T-FeAstV/ 18-T-FPV/ 18-T-FCoV plasmids. (**B**) Lane M, DNA marker (100 bp ladder plus); lane NC, negative control; lane 1, F81; lane 2, Escherichia coli; lane 3, Salmonella enterica; lane 4, rabies virus; lanes 5~9, 18-T-IAV, 18-T-*C. felis*, 18-T-FeLV, 18-T- FHV-1 and 18-T-FCV, respectively; lane 10, mixed standard 18-T-AIV/ 18-T-*C. felis*/ 18-T-FeLV/ 18-T- FHV-1/ 18-T-FCV plasmids.

We used the feline F81 cell line and other pathogens (Salmonella enterica, rabies virus and Escherichia coli) as templates to verify the specificity of the mPCR. No amplicons were observed in the lanes for the negative controls, Salmonella enterica, rabies virus, and Escherichia coli, while the lanes for FCoV, FeAstV, FPV and FeKoV (Figure 2A) and FCV, FHV-1, FeLV, *C. felis* and IAV (Figure 2B) showed amplification of specific bands.

### 3.3. Sensitivity of the mPCR Methods

**Figure 3:**
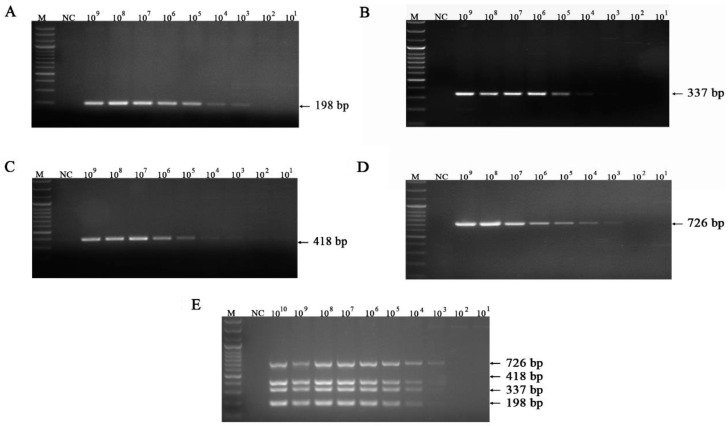
Sensitivity for FeKoV, FPV, FeAstV, and FCoV. (**A**) Sensitivity for FeKoV; (**B**) sensitivity for FPV; (**C**) sensitivity for FeAstV; (**D**) sensitivity for FCoV; (**E**) sensitivity for FeKoV/FPV/FeAstV/FCoV; lane M, DNA marker (100 bp ladder plus); lane NC, negative control.

**Figure 4:**
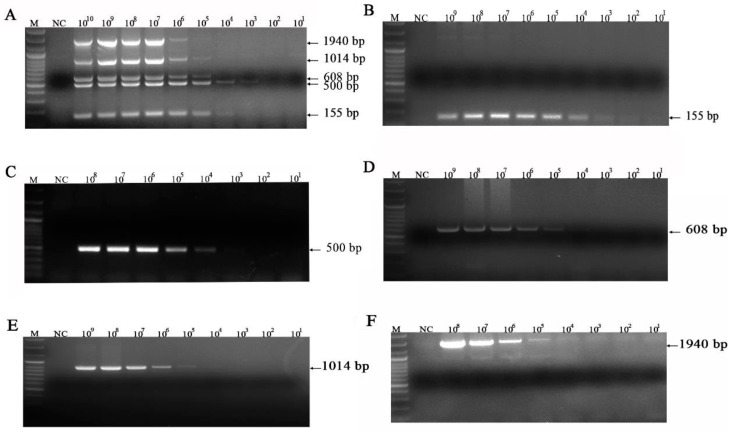
Sensitivity for FCV, FHV-1, FeLV, *C. felis* and IAV. (**B**) Sensitivity for IAV; (**C**) sensitivity for *C. felis*; (**D**) sensitivity for FeLV; (**E**) sensitivity for FHV-1; (**F**) sensitivity for FCV; (**A**) sensitivity for IAV/*C.felis*/FeLV/ FHV-1/FCV; lane M, DNA marker (100 bp ladder plus); lane NC, negative control.

The detection limits for FeKoV, FPV, FeAstV, and FCoV were 10^3^ (Figure 3A), 10^4^ (Figure 3B), 10^3^ (Figure 3C), and 10^3^ (Figure 3D) copies/µL, respectively. The detection limits for IAV, *C. felis*, FeLV, FHV-1 and FCV were 10^3^(Figure 4B), 10^4^ (Figure 4C), 10^4^ (Figure 4D), 10^5^ (Figure 4E) and 10^5^ (Figure 4F) copies/µL, respectively. The simultaneous detection limit of the mPCR method for FeKoV, FPV, FeAstV and FCoV was 10^3^ copies/µL (Figure 3E), while the simultaneous detection limit for IAV, *C. felis*, FeLV, FHV-1 and FCV (Figure 4A) was 10^3^ copies/µL.

### 3.4. Examination of Clinical Samples

**Figure 5:**
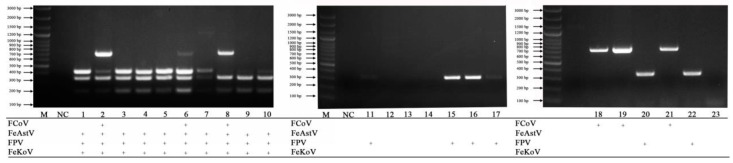
Examination of anal clinical samples. Lane M, DNA marker (100 bp ladder plus); lane NC, negative control; lanes 1–23, anal swab clinical samples.

**Figure 6:**
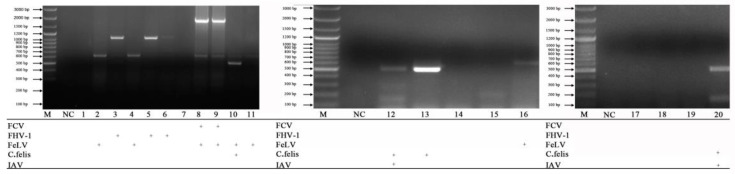
Examination of nasal clinical samples. Lane M, DNA marker (100 bp ladder plus); lane NC, negative control; lanes 1–20, nasal swab clinical samples.

The mPCR for detecting FeKoV, FPV, FeAstV and FCoV (Figure 5) was tested on 23 anal swab clinical samples. The mPCR for detecting FCV, FHV-1, FeLV, *C. felis* and IAV (Figure 6) was tested on 20 nasal swab clinical samples. When the two mPCR methods were applied to clinical samples, nine pathogens were simultaneously detected.

## 4. Discussion

Primer design is the key to the successful construction of mPCRs. First, each primer has good specificity for its own target fragment, and there should be no mismatch between primers. Due to the presence of multiple primer pairs, there is an increased likelihood of primer dimer formation [27,28]. When designing primers, this nonspecific binding should be minimized. The size of the amplified product is also one of the influencing factors, and the length of each product should be easy to distinguish. Second, the primer should not form a hairpin structure, as the formation of a hairpin structure will affect the combination between the primer and the template. In the mPCR method of our study, the annealing temperatures of the primers were similar, the specificity of the primers was good, and there was no mismatch between primers. The amplification products of FCV, FHV-1, FeLV, *C. felis*, IAV, FCoV, FeAstV, FPV and FeKoV were easily distinguishable. According to the experimental results, the established mPCR detection method generally performed well in terms of specificity, sensitivity and clinical detection. However, the sensitivity of FHV-1 and FCV detection is relatively low, and future optimization of mPCR is needed to increase the sensitivity of detection for these two pathogens.

The key to mPCR testing is to avoid cross-contamination. Partitioning each test can effectively reduce and avoid cross-contamination. In addition, premixing the reaction system in advance can effectively reduce cross-contamination. This study uses PCR premix. Only mixed primers, ddH2O and templates were added during the test. The test operation is simple, which can reduce the probability of cross-contamination during the addition of samples.

The mPCR system contains multiple primers, which can be used to identify by multiple pathogens, saving much cost and time for laboratory diagnosis. In the field of infectious diseases, most clinical cases have coinfections. It is difficult to diagnose and distinguish these pathogens according to clinical symptoms. The advantage of the mPCR method is that it can identify multiple diseases at the same time, so it is suitable for the diagnosis of coinfection. Clinical survey data showed that these pathogens are prevalent in cats.

In a molecular survey of feline infectious diseases in China from 2016 to 2019, 1060 (79.9%) of 1326 feline clinical samples tested positive for at least one virus. The positivity rates for FeLV, FPV, FHV-1, FCV and FIPV were 59.6%, 19.2%, 16.3%, 14.2% and 0.5%, respectively [29]. Therefore, the rapid and accurate identification of these pathogens is of great significance for their prevention and control. In addition, FeAstV and FeKoV, as emerging viruses, need to be monitored and epidemiologically investigated to further confirm the relationship between their infections and feline intestinal diseases.

In this study, we included FeLV and IAV in mPCR detection. FeLV is prevalent in cats worldwide. FeLV can be transmitted through oropharyngeal secretions [30]. Therefore, it is practical to detect FeLV in nasal swabs. FeLV infection can cause immunosuppression. If a cat is not treated in time, secondary infection can occur. A recent study showed that immunocompromised FeLV-positive cats are susceptible to SARS-CoV-2 infection [31]. At the current time of the coronavirus pandemic, it is possible that FeLV-positive cats act as a reservoir for new SARS-CoV-2 variants. Therefore, the rapid diagnosis of FeLV is of great significance. Regarding IAV, studies have shown that cats are susceptible to IAV. To date, a variety of subtypes of IAV have been isolated from cats, including H1N1, H3N2, H5N1, H5N6 and H7N2 [6,8,9,10,32]. In the report of H7N2 IAV, one veterinarian who had contact with the infected cat contracted the virus and showed mild respiratory symptoms before recovery. This evidence indicates that cats may act as an intermediate host during the transmission of IAV to humans or other mammals. The number of cats worldwide is increasing rapidly, and new recombinant IAV may infect cats and cause a risk of zoonotic infection. This situation should arouse elevated public health concern, so it is necessary to monitor IAV in cats.

## 5. Conclusions

In summary, the two newly established mPCR methods provide available, specific, sensitive and inexpensive tools for detecting pathogens (FCV, FHV-1, FeLV, *C. felis*, IAV, FCoV, FeAstV, FPV and FeKoV). In this study, we firstly included FeLV and IAV in the mPCR method to provide detection tools for assessing their prevalence and coinfection with other pathogens. This study provides an efficient tool for clinical diagnosis and laboratory epidemiological monitoring.

## Figures and Tables

**Table 1:** Primers used for mPCRs.

Pathogen	Primer Name	Primer Sequence (5′-3′)	Target Gene	PCR Products
FCV	FCV-F	AACCTGCGCTAACGTGCT	*VP1*	1940 bp
	FCV-R	TGWATTCCCATGTAGGAGGC		
FHV-1	FHV-1-F	TACTTCAAGCCTTACGACCACG	*gD*	1014 bp
	FHV-1-R	GATCGAGACCTCTTTACCCTCA		
FeLV	FeLV-F	TGGCACAGGTAAAGCAAGTT	*gPr80*	608 bp
	FeLV-R	CGTGTCCACCAARAARGTCA		
*C.felis*	*C.felis*-F	TTGTCGGATTGATTGGTCTT	*ompA*	500 bp
	*C.felis*-R	AGTTGGGTTCCAGGTTGTTA		
IAV	IAV-F	CAGAGACTKGAARRTGTCTTTGC	*M*	155 bp
	IAV-R	CTACGCTGCAGTCCTCGCTC		
FCoV	FCoV-F	GATGGAGTMTTCTGGGTTGC	*N*	726 bp
	FCoV-R	TTCCTCAGGCTTCTTATCAG		
FeAstV	FeAstV-F	GAAATGGACTGGACACGTTATGA	*ORF1ab*	418 bp
	FeAstV-R	GGCTTGACCCACATGCCGAA		
FPV	FPV-F	AAGACGTGCAAGCGAGTCC	*VP2*	337 bp
	FPV-R	GAGCGAAGATAAGCAGCGTAA		
FeKoV	FeKoV-F	CCTCTTTYCTTCGGGACTTTTA	*polyprotein*	198 bp
	FeKoV-R	ACCACATCACTGAYTGTTCGTA		

## Data Availability

Not applicable.
